# Increased SEMA6B expression as a potential prognostic and immune cell infiltration biomarker in thyroid cancer patients

**DOI:** 10.18632/aging.204691

**Published:** 2023-05-05

**Authors:** Qinghua Lei, Yunha Wang, Junhua Li, Shanshan Wang, Yanyan Hu, Lihua Duan, Yanfei Huo, Yiping Wu, Hongzhou Liu

**Affiliations:** 1 Department of Endocrinology, First Hospital of Handan City, Congtai, Handan 056002, Hebei Province, China; 2 Department of Endocrinology, The First Medical Center, Chinese PLA General Hospital, Haidian, Beijing 100853, China; 3 Department of Ultrasound Medicine, Handan Central Hospital, Congtai, Handan 056008, Hebei Province, China; 4 Department of Neurology, Handan Central Hospital, Congtai, Handan 056008, Hebei Province, China; 5 Physical Examination Center, Handan Central Hospital, Congtai, Handan 056008, Hebei Province, China

**Keywords:** thyroid cancer, SEMA6B, prognostic, biomarker, THCA

## Abstract

Background: Even today, thyroid cancer (THCA) remains an important threat to global health. For THCA patients, differentiated thyroid cancer is the most commonly identified pathological subtype, and those diagnosed with papillary thyroid cancer generally have good overall prognosis. For poorly differentiated subtype THCA, patients have aggressive disease course, higher risk of distant organ metastasis and inferior overall prognosis.

Methods: RNA-seq data from TCGA and GTEx databases are collected and analyzed via R. The correlation between SEMA6B expression level and pathological as well as clinical parameters of THCA patients was respectively investigated. Gene expression profiling and subsequent functional clustering analysis was the performed utilizing GSEA. The receiver operating characteristic (ROC) curve was utilized to evaluate the diagnostic value of SEMA6B expression.

Results: Increased SEMA6B expression was characteristic in THCA tumor samples and was associated with specific pathologic and clinical features for TCHA patients. Univariate and multivariate analysis indicated that SEMA6B was independent predictive marker for THCA patients’ prognosis. Gene expression profiling and functional clustering analysis suggested that SEMA6B high-expression was related with increased expression of multiple signal pathways and signatures of multiple immune cell infiltration.

Conclusions: In this study, through bioinformatic analysis and clinical data investigation, we demonstrated the potential value of SEMA6B as diagnostic and prognostic marker in THCA patient treatment.

## INTRODUCTION

As described in recent epidemiological study, thyroid cancer (THCA) stands as the seventh most common cancer in females from the United States. In 2021, over 40000 new cases were estimated to occur in both sexes [1]. For the pathological feature of THCA, differentiated thyroid cancer is the most commonly identified subtype of thyroid cancer, consisting more than 90% of total cases [2]. And its pathological cellular origin has been proved as thyroid follicular epithelial cells. Among all well-differentiated thyroid cancer, patients with papillary thyroid cancer generally have good overall prognosis [3]. However, poorly differentiated THCA subtypes are aggressive diseases and have higher risk of distant organ metastasis and significant inferior prognosis. Therefore, it is of clinical significance to fully uncover the underlying molecular mechanism in THCA metastasis and drug-resistance.

Through sequencing analysis on THCA samples researchers have unveiled the genetic basics for the pathogenesis of THCA. It has been suggested that mutations of genes involving crucial signaling pathways such as MAPK/RAS are characteristics in THCA patients [4]. Besides, chromosomal translocation is also frequently detected events in THCA pathogenesis, which is associated with the formation of a series of oncogenic fusion genes, including PAX8-PPARγ, ALK rearrangements, BRAF rearrangement, etc. [5, 6] Class 6 Semaphorin B (SEMA6B), is a transmembrane protein family member of Semaphorin, has been shown to participate in the pathogenesis of several malignancies [7, 8].

The roles of SEMA6B in different tumors have been reported. The SEMA6B gene was strongly downregulated in breast cancer tissues [8]. It has also been shown that SEMA6B modulated malignant biological behavior, including invasion, migration and adhesion, in gastric cancer [7]. Overexpression of SEMA6B indicated poor prognosis in colorectal cancer [9]. SEMA6B expression was decreased in two types of glioblastoma cell lines [10], and silencing SEMA6B expression could inhibit glioblastoma formation [11]. However, the exact role of SEMA6B in THCA has not been fully uncovered to date. Therefore, in the present study, we aimed to utilize clinical samples RNA sequencing (RNA-seq) data collected from a bioinformatic platform to investigate the role of SEMA6B in THCA, as well as its underlying mechanism in THCA progression and metastasis.

## RESULTS

### Increased SEMA6B was associated with clinical feature of THCA patients

**Figure 1:**
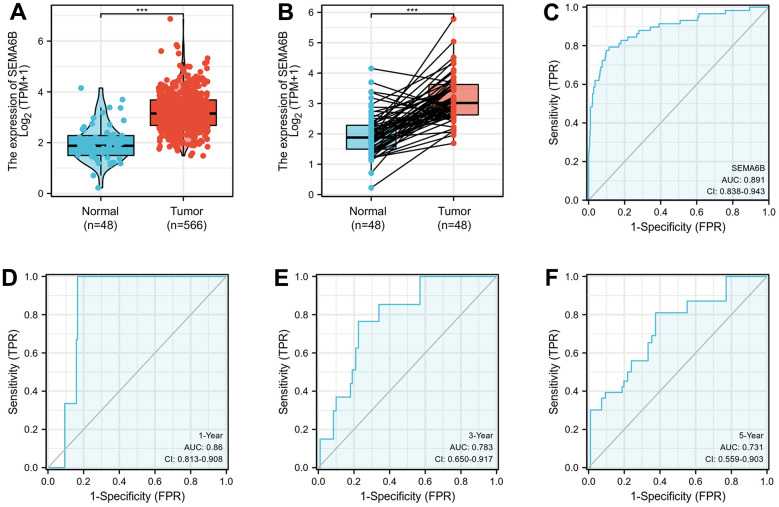
(**A**) RNA sequencing data analysis on SEMA6B mRNA expression differences between normal and thyroid cancer (THCA) tissues. (**B**) RNA sequencing validation on SEMA6B mRNA expression in paired samples of THCA and normal thyroid tissues. Data were retrieved from publicly available online platform GTEx (**C**–**F**) ROC curve for SEMA6B in THCA samples.

In this study, we compared the expression of SEMA6B in paired adjacent normal tissue and tumor samples from THCA patients. Results indicated that compared with adjacent normal tissues, THCA tumor samples demonstrated significantly higher level of SEMA6B (Figure 1A). Subsequently, validation experiment utilizing RNA-seq data from GTEx database also suggested consistent results (Figure 1B). Then receiver operation curve (ROC) analysis was performed to investigate the value of diagnostic prediction value of SEMA6B expression level. Results suggested that for THCA samples and normal samples, the area under the curve (AUC) was 0.891 (Figure 1C), further study indicated that SEMA6 expression also showed promising predictive value in THCA patients with different overall survival. As AUCs for SEMA6B expression in THCA patient subgroups with 1-year/3-year/5-year survival were 0.86, 0.783 and 0.731 respectively (Figure 1D–1F).

### Association between SEMA6B expression and clinical features of THCA patients

**Figure 2:**
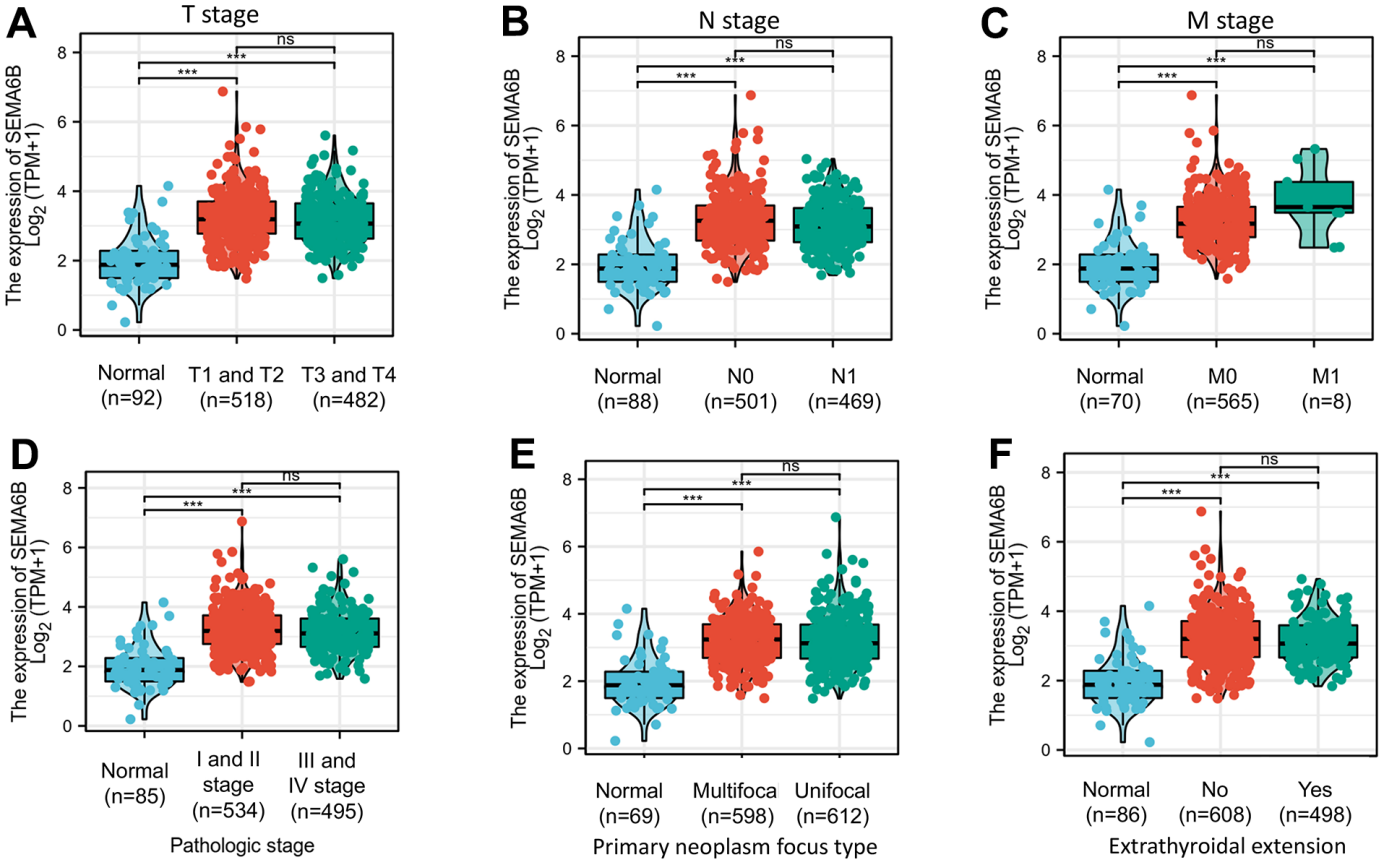
Box plot evaluating SEMA6B mRNA expression differences among THCA patients with different clinical characteristics including disease stages (TNM staging system) (**A**–**C**), tumor pathologic stages (**D**), primary neoplasm focus type (**E**) and state of extrathyroidal extension (**F**).

Then, we conducted further investigation on the association between SEMA6B and clinical features in THCA patients (n=502) collected from TCGA database. The basic clinical characteristics of the cohort was shown in Table 1. Surprisingly, result indicated that the expression of SEMA6B showed no significantly differences between patient subgroups of different clinical stage (T1/2 VS T3/4) (Figure 2A), lymph node invasion (N0 VS N1) (Figure 2B) and distant organ metastasis (M0 VS M1) (Figure 2C). Moreover, subsequent analysis also indicated that SEMA6B expression exhibited no significant differences between THCA patient groups with different pathologic stages (stage I/II VS stage III/IV) (Figure 2D), primary neoplasm focus type (multifocal VS unifocal) (Figure 2E) and status of extrathyroidal extension (Figure 2F).

**Table 1:** Basic clinical characteristic of thyroid cancer patients involved in this study.

**Characteristic**	**levels**	**Overall**
n		502
T stage, n (%)	T1	143 (28.6%)
	T2	164 (32.8%)
	T3	170 (34%)
	T4	23 (4.6%)
N stage, n (%)	N0	229 (50.7%)
	N1	223 (49.3%)
M stage, n (%)	M0	282 (96.9%)
	M1	9 (3.1%)
Pathologic stage, n (%)	Stage I	281 (56.2%)
	Stage II	52 (10.4%)
	Stage III	112 (22.4%)
	Stage IV	55 (11%)
Extrathyroidal extension, n (%)	No	331 (68.4%)
	Yes	153 (31.6%)
Primary neoplasm focus type, n (%)	Multifocal	226 (45.9%)
	Unifocal	266 (54.1%)

**Figure 3:**
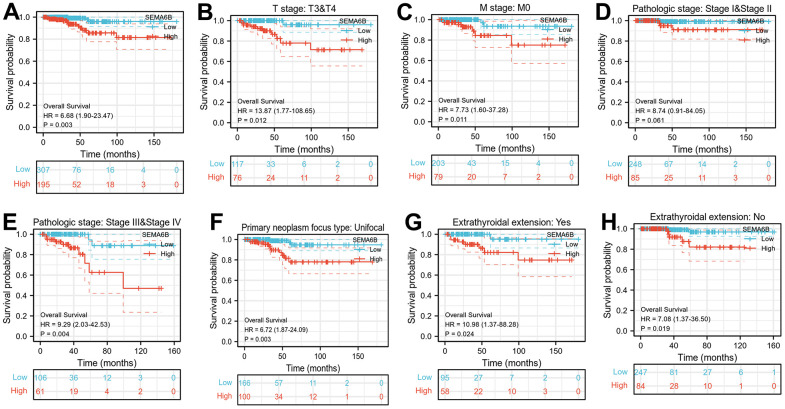
(**A**) Kaplan-Meier curve indicating overall survival comparison between THCA patient subgroup with high/ low SEMA6B mRNA expression; (**B**–**H**) Overall Survival comparison between THCA Patients Subgroup with SEMA6B high/low expression, patient subgroups were divided by TNM stages (**B**, **C**), Pathologic stages (**D**, **E**), Primary tumor focus type (**F**), Extra thyroidal extension (**G**, **H**) (n=502).

Next, we aimed to explore the prognostic value of SEMA6B expression in THCA patients. Through collected prognostic data comparison between THCA patients with high/low SEMA6B expression, significant differences of overall survival were identified. In detail, patients with high SEMA6B expression level suffered notably inferior prognosis compared with those counterparts with low SEMA6B expression (Figure 3A). In addition, in subgroup analysis of patients with advanced clinical stage (T3 and T4), without distal organ metastasis (M0), with advanced pathological stage (stage III/IV), with unifocal neoplasm pathological type, with or without extrathyroidal extension, SEMA6B high-expressed patients suffered significantly inferior overall prognosis compared with SEMA6B low-expressed patients (Figure 3B, 3C, 3E–3H). However, the only exception was noted in patients with early clinical stages (stage 1-2), as these patients’ prognosis demonstrated no significant differences.

### Diagnostic and risk predictive value of SEMA6B expression

**Figure 4:**
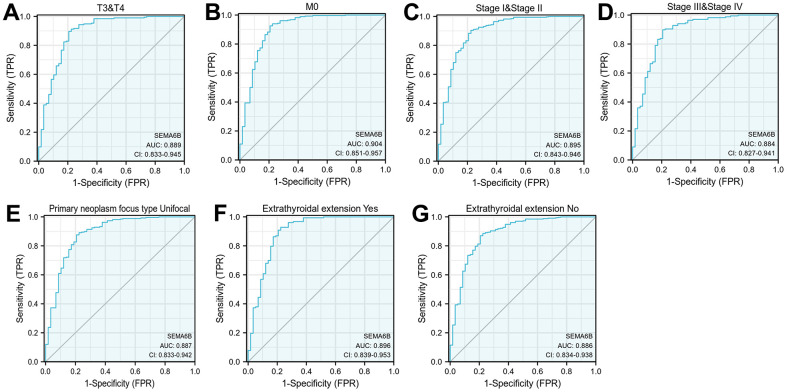
**Diagnostic value analysis of SEMA6B expression in THCA.** ROC curve indicated the sensitivity and specificity of SEMA6B expression in THCA subgroup differentiation including TNM staging (**A**, **B**), pathologic staging (**C**, **D**), primary neoplasm focus (**E**), extrathyroidal extension (**F**, **G**) (n=455).

Subsequently, diagnostic value of SEMA6B expression in THCA patient subgroups were investigated utilizing ROC curve analysis. As shown in Figure 4A–4D, the expression of SEMA6B exhibited relatively high diagnostic value in predicting subgroups of patients with T3/T4 (AUC 0.889, 95%CI 0.833-0.945), M0 (AUC 0.904, 95% CI 0.851 – 0.957), Stage I/II (AUC 0.895, 95% CI 0.843-0.946) or stage III/IV (AUC 0.884, 95%CI 0.827 – 0.941) status. SEMA6B expression also showed value in patients with unifocal primary neoplasm pathological type (AUC 0.887, 95%CI 0.833 – 0.942) and patients with or without extra-thyroid extensions (AUC 0.896 / 0.886, 95%CI 0.839 – 0.953 / 0.834 – 0.938) (Figure 4E–4G).

**Figure 5:**
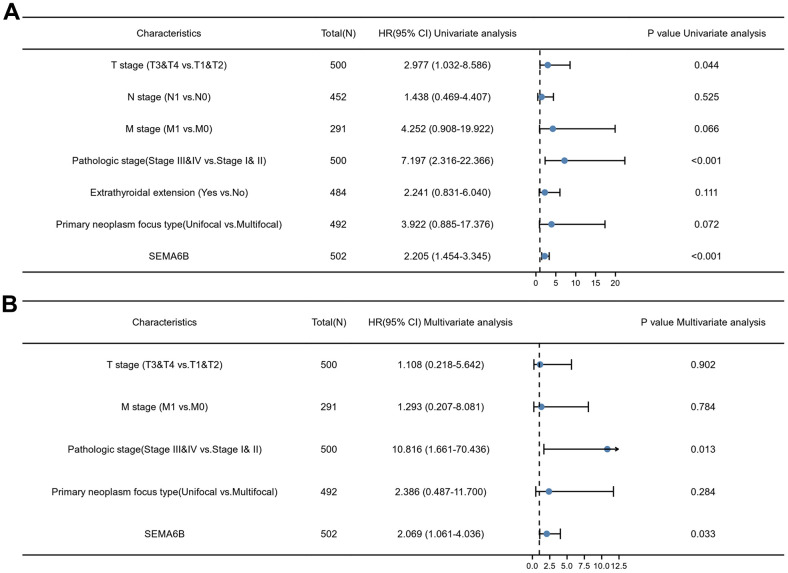
**Univariate and multivariate Cox regression analyses of multiple clinical characteristics associated with overall survival.** Inspected clinical characteristics includes TNM stages, pathologic stages, primary neoplasm focus type, extrathyroidal extension and SEMA6B expression level.

**Figure 6:**
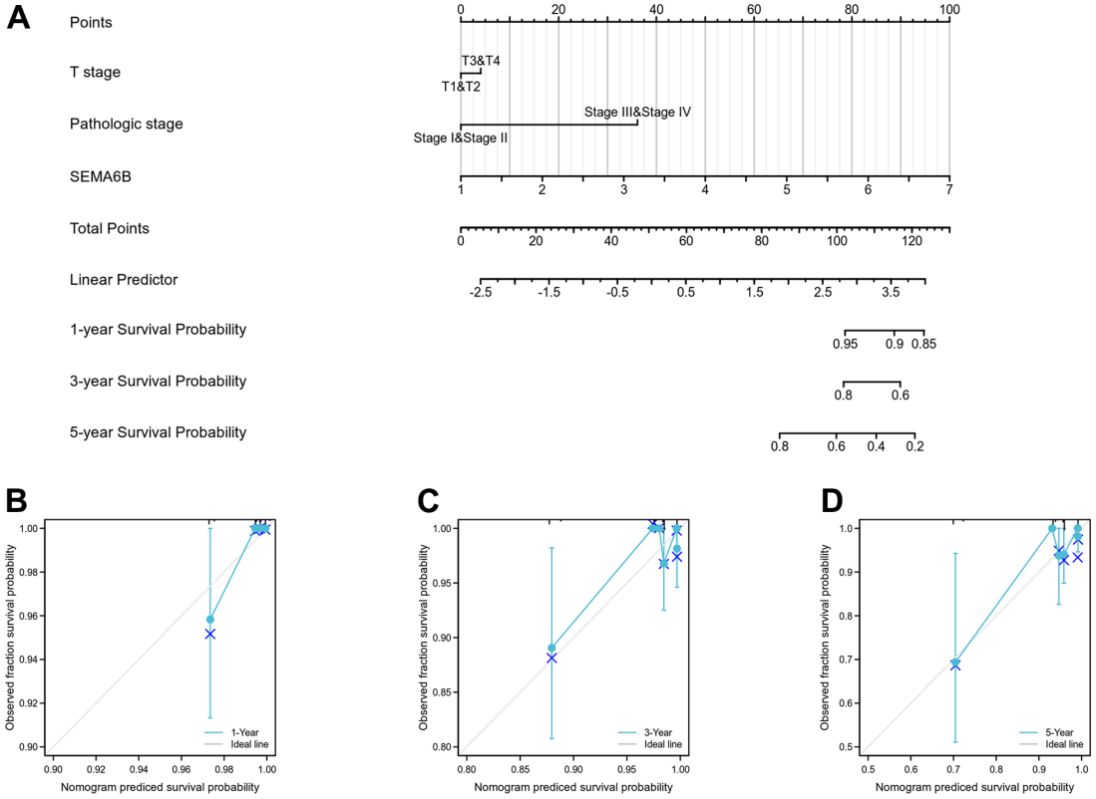
**Nomogram curve for predicting probability of patients with 1, 3 and 5 year overall survival (OS).** Clinical stage, pathologic stage, SEMA6B expression was analyzed to predict OS (**A**), 1-year OS in THCA population was predicated (**B**), 3-year OS in THCA population was predicated (**C**), 5-year OS in THCA population was predicated (**D**).

In addition, univariate and multivariate Cox regression analyses were performed to identify clinically relevant risk factors associated overall survival of THCA patients. As shown in Figure 5, in univariate analysis, we discovered that advanced pathologic stage (stage III/IV) (p<0.001) and SEMA6B expression (p<0.001) were significantly associated with inferior overall survival of THCA patients. Moreover, multivariate analysis also provided consistent results, indicated that advanced pathological stages and SEMA6B expression were independent predictor for inferior prognosis of THCA patients. Besides, the nomogram including clinical stage, pathologic stage, SEMA6B expression was used to predict probability of the 1, 3, 5-year OS in the THCA population (Figure 6A–6D).

### Expression profiling analysis suggested distinct SEMA6B-related molecular and immune pathway

**Figure 7:**
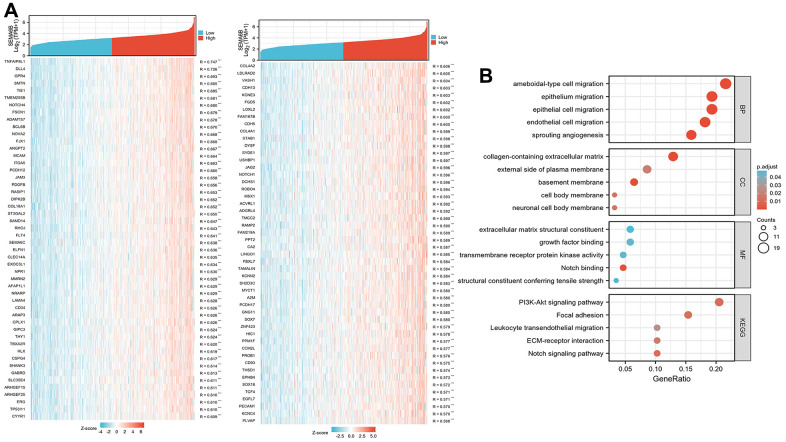
**Differentially expressed gene profiling between group SEMA6B-high expression and group SEMA6B-low expression.** Differentially expressed genes were ranked by correlation factor R (**A**), and the gene list was further clustered via GO/KEGG analysis (**B**).

In order to delineate the potential SEMA6B-associated gene regulatory network, we further analyzed GTEx RNA-seq data between group SEMA6B-high expression and group SEMA6B-low expression. As shown in Figure 7A, top up-regulated and down-regulated genes were listed. Further gene-set enrichment (GSEA) analysis was performed and results showed in Figure 7B indicated that several gene functional clusters were significantly enriched in SEMA6B high-expression THCA patients, including cellular migration, adhesion and PI3K-Akt pathways.

**Figure 8:**
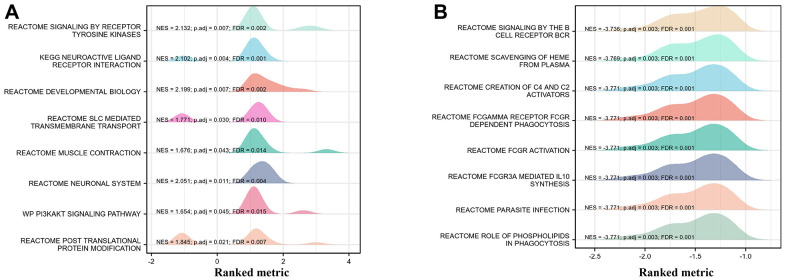
**GSEA analysis on functional cluster of differentially expressed genes in THCA cancer cells with high/low SEMA6B expression level.** Tyrosine kinase pathway and neuroactive ligand receptor pathway were significantly up-regulated (**A**), BCR-related signaling, C4/C2 activator as well as cellular phagocytosis were significantly down-regulated (**B**).

Following reactome pathway analysis also suggested that tyrosine kinase pathway (NES 2.132, FDR 0.002), Neuroactive ligand receptor pathway (NES 2.102, FDR 0.001) were significantly up-regulated reactome pathways (Figure 8A). Meanwhile, BCR-related signaling (NES -3.736, FDR 0.001), C4/C2 activator (NES -3.771, FDR 0.001) as well as cellular phagocytosis (NES -3.771, FDR 0.001) were significantly down-regulated reactome pathways (Figure 8B).

**Figure 9:**
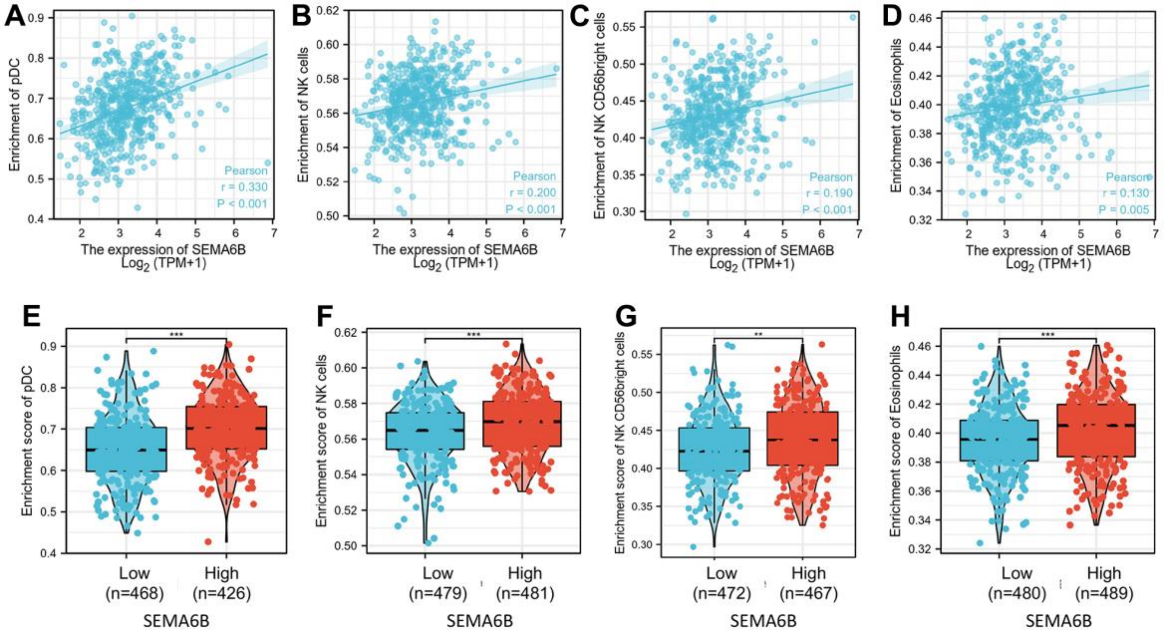
**Correlation of enrichment of infiltrated immune and myeloid cell in tumor tissue and SEMA6B expression level in clinical samples of THCA patients.** Innate immune cells investigated include DC cells (**A**), NK cells (**B**, **C**), and eosinophils (**D**), and percentage differences of each kind of the infiltrated cells between THCA tumor sample groups with high/low SEMA6B expression level were subsequently compared (**E**–**H**).

**Figure 10:**
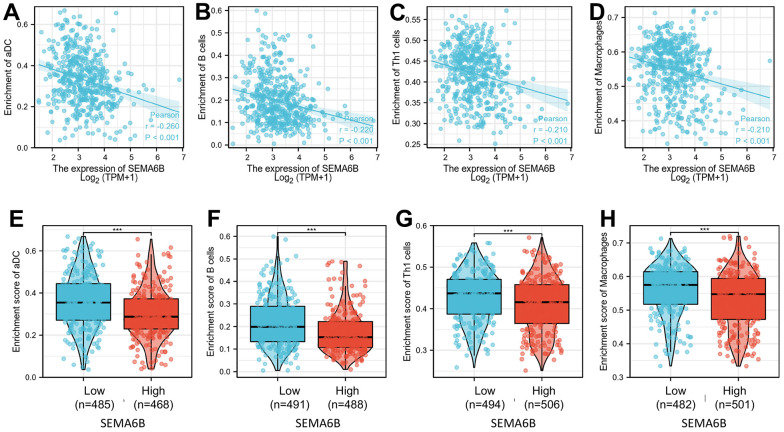
**Correlation of enrichment of infiltrated immune cells and macrophage in tumor tissue and SEMA6B expression level in clinical samples of THCA patients.** Immune cells investigated include aDC cells (**A**), B cells (**B**), Th1 cells (**C**) and macrophages (**D**). Differences of each infiltrated immune cells between THCA tumor sample groups with high / low SEMA6B expression level were subsequently compared (**E**–**H**).

**Figure 11:**
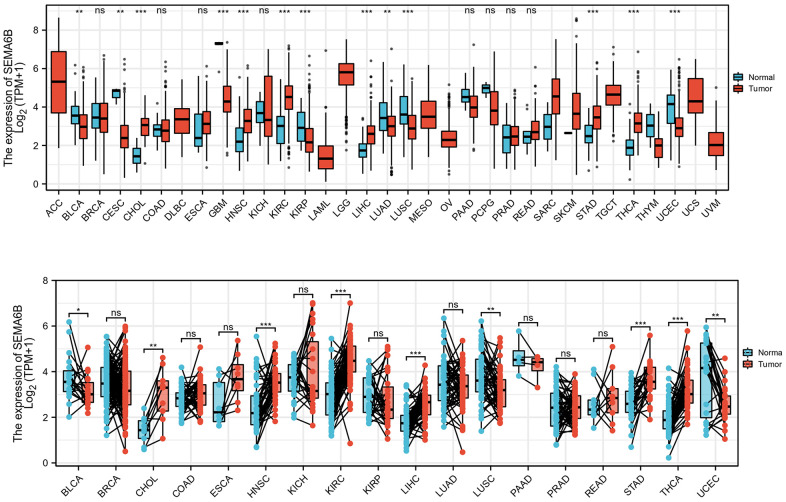
**Expression comparison of SEMA6B mRNA level in matched tumor and adjacent normal tissue from multiple malignancies.** SEMA6B mRNA level was calculated from RNA-seq data from tumor sample and match normal tissue in TCGA database (n=17 for normal tissues, n=18 for tumor tissues).

To explore the immune infiltration signature in SEMA6B high THCA patients, ssGSEA analysis utilizing spearman correlation was conducted. As shown in Figure 9A–9D, experiment results indicated that SEMA6B high expression was significantly associated with pDC, NK cells eosinophils infiltration signature in the tumor microenvironment. In addition, comparison of enrichment scores also provided consistent results that SEMA6B high-expression tumor samples exhibited significantly higher enrichment scores involving pDC\NK cells\eosinophils infiltrations (Figure 9E–9H). Likewise, we also demonstrated that SEMA6B high-expression was also significantly negatively related with aDCs\B cells\Th1 cells\macrophages infiltration signatures (Figure 10A–10D), and enrichment scores also validated our findings (Figure 10E–10H). Besides, by further investigating SEMA6B in multiple different malignancies, we also confirmed that SEMA6B high-expression is characteristic not only in THCA but also in clear cell renal carcinoma (KIRC) and stomach adenocarcinoma (STAD) (Figure 11).

### Knockdown of SEMA6B inhibited the cell proliferation, migration, invasion of thyroid cancer cells, but promoted apoptosis

**Figure 12:**
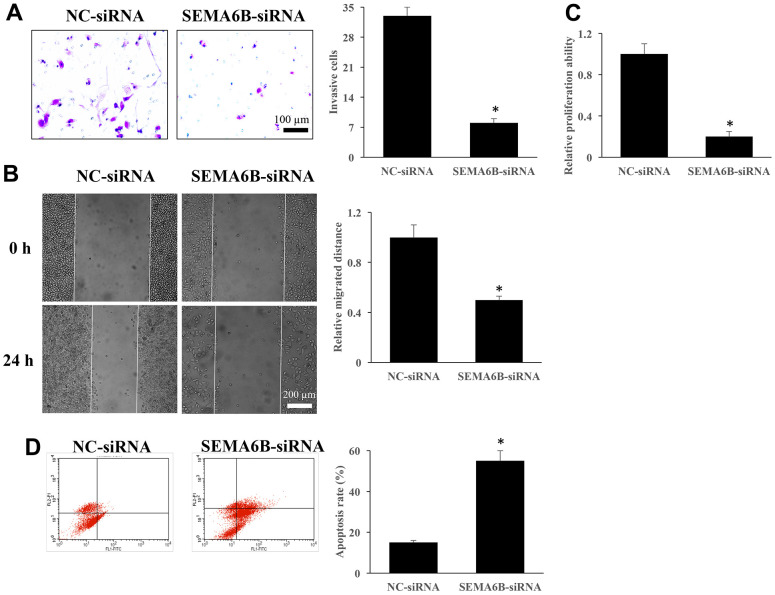
**Knockdown of SEMA6B inhibited the cell proliferation, migration, invasion of thyroid cancer cells, but promoted apoptosis.** The influence of SEMA6B on thyroid cancer cells invasion (**A**). The influence of SEMA6B on thyroid cancer cells migration (**B**). The influence of SEMA6B on thyroid cancer cells proliferation (**C**). The influence of SEMA6B on thyroid cancer cells apoptosis (**D**). *<0.05 compared with group NC-siRNA. These experiments were repeated at least 3 independent times.

The influence of SEMA6B-siRNA on thyroid cancer cells viability was investigated. We found that SEMA6B-siRNA markedly suppressed the invasion (Figure 12A), migration (Figure 12B), and proliferation (Figure 12C) of thyroid cancer cells. In addition, the tumor cell apoptosis was promoted by knocking down SEMA6B (Figure 12D).

## DISCUSSION

The present study, through bioinformatic analysis on GTEx and clinical sample RNA-seq datasets, provided evidence that SEMA6B may be a potential informative biomarker for patients with THCA, suggesting its diagnostic and prognostic value, not only in general tumor-bearing population, but also in subgroup of patients with specific disease stage, pathological type and metastatic state. The results of the present study are consistent with previous findings based on TCGA datasets analysis and cell model experiments [12]. Further univariate and multivariate analyses confirmed that SEMA6B expression was an independent prognostic marker, indicating lower survival for patients with THCA. The current results suggest the potential clinical utility of a SEMA6B expression test as a practical strategy to differentiate patients with high risk of disease progression, distal organ metastasis and lower survival.

A previous study indicated that high SEMA6B levels were associated with adverse prognosis of patients with colorectal cancer. SEMA6B could be considered as a novel prognostic biomarker for colorectal cancer [9]. The role of SEMA6B in colorectal cancer is similar to its function in THCA described in the present study. However, SEMA6B gene products were strongly downregulated in breast cancer tissues [8]. These data suggest that the role of SEMA6B in different types of tumors is complex.

The diagnostic value of SEMA6B in subgroups of patients with THCA was validated through ROC curve analysis in the present study. The level of SEMA6B presented a relatively high diagnostic value in predicting subgroups of patients with T3/T4, M0 and stage I/II or III/IV status, and all the AUC values were >0.85. The diagnostic value of SEMA6B in colorectal cancer was also reported, but the values of AUC ranged from 0.639 to 0.759 [9], which were lower than those revealed in the current study.

Further analysis of RNA-seq data between SEMA6B high/low groups of patients with THCA identified significantly differentiated genes and functional gene clusters associated with SEMA6B high expression. A previous study indicated that SEMA6B exerted a tumorigenic effect in thyroid carcinoma partly by activating the Notch signaling pathway [12]. The present results indicated that SEMA6B high expression was significantly associated with genes involved in cellular migration and the Notch/PI3K/Akt signaling pathway, suggesting a potential molecular interaction between SEMA6B and the PI3K signaling pathway in the promotion of tumor cell migration.

The novelty of the current study is that it demonstrated the association between SEMA6B and the PI3K/Akt signaling pathway. This result was consistent with previous findings reporting that knocking down SEMA6B significantly reduced Notch1 and cyclin D1 expression, and notably reduced thyroid tumor cell migration and viability (9). Dysregulation of the Notch signaling pathway has been confirmed to be involved in enhanced tumor cell proliferation, metastasis and epithelial-mesenchymal transition of multiple malignancies [13–16]. Activation of the Notch signaling pathway is associated with inferior prognosis of breast cancer [17, 18]. Besides, Notch has also been shown to interact with the NF-κB and PI3K/Akt signal pathways. A previous study has indicated that Notch1 activation can trigger Wnt/β-catenin and NF-κB activation mediated by Akt phosphorylation, which enhances the invasive capabilities of glioblastoma [19]. Additionally, Notch prevents the dephosphorylation of the PI3K/Akt signaling pathway through protein phosphatase 2 A inhibition and PTEN activation, resulting in enhancement of tumor cell malignant biological behavior [20–22]. Based on the above findings, the interaction between SEMA6B, Notch1 and the PI3K/Akt signaling pathway should be investigated in the future, which may provide novel clues for treatment development for high-risk patients with THCA.

The present study also demonstrated a distinct feature of immune cell infiltration gene expression signatures identified by RNA-seq analysis. The current results suggested that the NK cell infiltration signature was significantly positively correlated with SEMA6B expression in patients with THCA, while adaptive immune cells, including Th1 and B cells, as well as macrophages were negatively correlated with SEMA6B expression. Previous studies on gene expression profiling [23, 24] have also suggested several immune-related gene signatures that may have clinical value in differentiating patients with THCA with inferior prognosis. Consistently, the authors also suggested that the patient subgroup with higher risk was associated with lower infiltration levels of B cells, CD4^+^ Th1 cells and macrophages. It was well established in previous studies that Th1 cells and macrophages infiltration exerts antitumor effects via antigen recognition [25–27]; therefore, further exploration of the impact of SEMA6B on those immune cell infiltrations in patients with THCA it is worthy. Moreover, as immunotherapy has shown a promising future in multiple malignancies, including THCA [28, 29], whether patients with THCA exhibiting SEMA6B high expression could benefit from immunotherapy deserves future investigation.

## CONCLUSIONS

In conclusion, the current study, through high-throughput bioinformatic analysis, demonstrated that SEMA6B high expression was characteristic in patients with THCA. Subsequent analysis indicated that SEMA6B high expression was correlated with distinct pathological and clinical features of patients with THCA. Further investigation suggested that SEMA6B-high expression was associated with a distinct pattern of upregulation of multiple functional signaling pathways, including Notch/RAS/MAPK, as well as unique immune-cell infiltration gene signatures. The current results indicated the potential value of SEMA6B as a diagnostic and prognostic marker in the treatment of patients with THCA.

## MATERIALS AND METHODS

### Clinical sample dataset collection

All transcriptome RNA-seq data and the related clinical information of THCA patients were collected from TCGA database (https://tcga-data.nci.nih.gov/tcga/). Normal thyroid tissues data were obtained from GTEx datasets (https://commonfund.nih.gov/GTEx). All methods were carried out in accordance with the Declaration of Helsinki. Informed consent was obtained from all participants. In this study, “TNM” stage method was used. “T” stage refers to the situation of primary tumor. Based on the tumor volume, the depth of invasion, and the range of adjacent tissue involvement, T1, T2, T3, T4 were used to express the situation of primary tumor. “N” stage refers to the involvement of regional lymph nodes, and N0 indicates that the lymph nodes are not involved. With the increase of the degree and scope of lymph node involvement, N1 and N2 were used. “M” stage means distant metastasis, M0 indicates without distant metastasis, and M1 indicates with distant metastasis. Based on the situations of “TNM”, the tumors were divided into four stages, which were indicated by I, II, III, IV. During the analysis process, “TNM” and stage “I, II, III, IV” were used.

### Gene set enrichment analysis (GSEA)

GSEA was conducted to detect potential biological processes and pathways in the SEMA6B high-expression subgroup in the ‘cluster Profiler’ R package. The c2.cp.v7.2.symbols.gmt (Kyoto Encyclopedia of Genes and Genomes) and c5.all.v7.2.symbols.gmt (Gene Ontology) were acquired from the Molecular Signatures Database. Gene sets with |normalized enrichment score (NES)|>1, nominal (NOM) P<0.05 and false discovery rate (FDR) <0.05 were considered to indicate a statistically significant difference. The data from non-tumor tissues were obtained from GSEA.

### Immune cells infiltration of ssGSEA

Immune infiltration analysis of THCA was conducted utilizing single-sample gene set enrichment analysis (ssGSEA) in the “GSVA” R package. And the infiltration level of each kind of immune cell type from THCA clinical sample was calculated from related gene expression profile. Additionally, Spearman correlation was utilized to explore the association of immune cells infiltration with SEMA6B expression and Wilcoxon rank sum test was performed to investigate the correlation between infiltration level of different immune cell types and SEMA6B expression level.

### Cell invasion detection

CAL-62 cell line was used in this study. The NC-siRNA and SEMA6B-siRNA were designed and constructed by Biosyntech (Suzhou, China). 1×10^5^ cells were put to the upper chamber, and medium containing 10% FBS (Gibco, USA) were added to the lower chamber. PBS (Gibco, USA) was used to wash cells twice after 24 h incubation. A cotton swab was used to wipe off cells. Pure methanol was used to fix cells for 10 min. Cells were stained with 0.2% crystal violet for 10 min, and images were obtained using an inverted microscope. The invasive cells were calculated with Image J software.

### Cell migration measurement

The cells (3×10^5^/well) were seeded into a 6-well plate. Wound healing assay was performed with a 1 mL pipette tip. Then, the wound healing areas were recorded at 0 h and 24 h. The migrated distance was calculated with Image J software.

### Cell proliferation detection

The cells (1×10^5^/well) were plated into a 96-well plate and cultured for 24 h. CCK8 kit (Beyotime, Shanghai, China) was used to measure cell proliferation ability.

### Cell apoptosis detection

After digestion, the cells were collected. Cold PBS was used to wash cells for 3 times. The cells were centrifuged at 1500 g/min for 20 min. Cells were incubated with Propidium iodide (Beyotime, Shanghai, China) and Annexin V-FITC (Beyotime, Shanghai, China) in the dark for half hour. Cell cytometry was used to analyze cell apoptosis. Flow cytometry analysis (Agilent, Novocyte cytometer, USA) was used to analyze cell apoptosis. Flowjo™ software v11 was used for flow cytometry data analysis.

### Cell transfection

SEMA6B siRNA (50 nM) and control siRNA (50 nM) were designed and supplied by Santa Cruz Biotechnology, Inc. Cell transfection was performed with Lipofectamine (Thermo Fisher Scientific, Inc.). Cells were firstly seeded on 60-mm dish. After reaching 70% confluence, cell transfection was performed with Lipofectamine 2000 (Invitrogen, Carlsbad, CA, USA).

### Statistical analysis

The SEMA6B expression in patients with THCA was quantified and compared with matched normal thyroid tissues utilizing GTEx datasets. The median method of SEMA6B expression was chosen as cut-off value to differentiate patient subgroup with SEMA6B-high/SEMA6B-low expression level. The association between clinical features and SEMA6B expression in THCA patients was tested by Wilcoxon signed-rank test and logistic regression. The overall survival (OS) between the high and low SEMA6B expression patient groups were tested by the Kaplan–Meier analysis. The receiver operating characteristic (ROC) curve was utilized to evaluate the diagnostic value of SEMA6B expression, with the area under the ROC curve designated as the prediction level of diagnosis. Univariate and multivariate Cox analysis on TCGA-THCA patient cohort were performed to identify independent prognostic predictor for overall prognosis. Multivariate Cox analysis was further applied to verify the independent prognostic prediction value of SEMA6B expression, and a nomogram was produced to predict 1-, 3- and 5-year OS for THCA patients.

### Data availability statement

The data presented in this study are available in this article.
